# Report on the clinical outcomes of using postural drainage with intervertebral space as the main focus for managing thoracolumbar tuberculosis: Eight case reports

**DOI:** 10.1097/MD.0000000000041204

**Published:** 2025-01-03

**Authors:** Hao Xing, Jianhua Li, Zhengqi Chang

**Affiliations:** a Department of Orthopedics, 960th Hospital of PLA, Jinan, China.

**Keywords:** intervertebral spaces, percutaneous endoscopic debridement of lumbar lesions, postural drainage, thoracolumbar tuberculosis

## Abstract

**Rationale:**

Conservative treatment has shown limited effectiveness in managing thoracolumbar tuberculosis (TB) that extends to the intervertebral space, as antibiotics are unable to penetrate avascular intervertebral discs, while conventional surgery is known for its extensive trauma and slow healing process.

**Patient concerns:**

Infection of the thoracic and lumbar vertebrae with tuberculosis can lead to difficulties in treatment due to involvement of the intervertebral space.

**Diagnosis:**

The diagnosis of TB was confirmed through clinical manifestations, impact studies, and T-spot experiments.

**Interventions:**

A retrospective analysis was conducted on the clinical data of 8 patients with thoracolumbar spinal TB who underwent postural drainage focusing on the intervertebral space as a treatment from June 2012 to August 2019. The average duration of treatment was 7.75 ± 10.19 months. Among the patients, there were 2 cases of thoracolumbar and 6 cases of lumbar spine involvement, with 6 cases affecting a single segment and 2 cases involving 2 segments. The total number of affected vertebrae included 4 thoracic, 12 lumbar, and 2 sacral vertebrae. All patients received standardized quadruple antituberculosis treatment (HRZE scheme). Specific postural drainage paths and catheter placement locations were determined based on clinical imaging results, and percutaneous catheter placement was performed with the assistance of foraminoscopy. Erythrocyte sedimentation rate, C-reactive protein, Visual Analogue Scale score, American Spinal Cord Injury Association score, and Oswestry function index were statistically analyzed before, after postural drainage, and at the final follow-up.

**Outcomes:**

The average operation time was 44.38 ± 10.50 minutes, with a blood loss of 6.88 ± 2.59 mL. The average catheter drainage time was 13.25 ± 4.95 days, and the follow-up period ranged from 36 to 122 months. The average total drainage volume was 281.25 ± 167.69 mL. Significant improvements were observed in erythrocyte sedimentation rate, C-reactive protein, Visual Analogue Scale score, American Spinal Cord Injury Association score, and Oswestry functional index at 7 days after postural drainage and at the last follow-up compared to before postural drainage (*P* < .05). At the last follow-up, there were 8 cases with no recurrence reported among the patients.

**Lessons:**

The utilization of positional drainage in the intervertebral space, coupled with chemotherapy, has demonstrated encouraging clinical results and may be deemed appropriate for treatment.

## 1. Introduction

Tuberculosis (TB) of the spine and joint is one of the most common extrapulmonary TB, and spinal TB accounts for about 50% of bone and joint TB, of which the lower thoracic and lumbar spine are the most common sites, and the vertebral body and intervertebral space are the most common.^[1–3]^ Spinal TB often has (an) insidious onset and slow progression, with atypical early symptoms and a low positive rate of bacterial culture, which is easy to miss and misdiagnose. If spinal TB is delayed or treated improperly, it can easily lead to a higher disability rate. Therefore, once spinal TB is diagnosed, it must be treated regularly and systematically.^[4]^ At present, there is a lot of controversy about the choice of conservative treatment or surgical treatment. Patients with severe low back pain, spinal nerve dysfunction, giant paravertebral cold abscess formation, kyphosis and segmental instability should be treated with surgical treatment.^[5,6]^ However, with the advancement of surgical techniques, a series of minimally invasive surgeries have been applied to the treatment of spinal diseases, such as percutaneous endoscopic lumbar discectomy,^[7,8]^ unilateral biportal endoscopic technique,^[9,10]^ lateral lumbar interbody fusion,^[11,12]^ etc. The operation is quick and has little impact on the internal environment, expanding the indications for surgery, and early surgical intervention for some patients.

In the pathogenesis of spinal TB, the vertebral body is the first site to be invaded. At this stage, the effect of drug chemotherapy is good, and conservative treatment is feasible; as the disease progresses, the TB infection breaks through a natural chamber and invades the intervertebral space, which is a sign Tuberculosis has entered a new stage.^[5]^ At this time, *Mycobacterium tuberculosis* colonizes the intervertebral disc tissue without blood supply to form a biofilm, which further develops into a “bacteria nest,” and the effect of drug chemotherapy at this stage will be greatly reduced.^[13]^ *M tuberculosis* is centered in the intervertebral space, and if it continues to form a cold abscess and breaks through the natural chamber around it, it promotes the progression of the disease and enters the adjacent vertebral body, psoas muscle, spinal canal, etc^[14]^; if it is suppressed by chemotherapy drugs or the body’s immunity, the lesions are limited to the intervertebral space, which leads to the recurrence of this disease.

In view of the above reasons, we believe that the treatment of the intervertebral space is very important. Removing the “bacterial nests” that drain TB can not only reduce the bacterial load at the lesion site but also prevent bacterial reproduction from the root. We retrospectively analyzed a total of 51 TB patients who were treated from June 2012 to August 2019. Among them, 8 cases of thoracolumbar spinal TB were treated by postural drainage with the intervertebral space as the treatment center, and satisfactory clinical effects were achieved.

## 2. Materials and methods

### 2.1. Materials

Case inclusion criteria: (1) patients who were diagnosed with spinal TB by clinical symptoms, signs, imaging, and laboratory tests; (2) patients who underwent local anesthesia for “dissection and drainage of lesions centered on the intervertebral space.”

Case exclusion criteria: (1) spinal infection other than TB was excluded; (2) obvious space destruction, high risk of spinal instability; (3) significant symptoms of spinal nerve compression; (4) significant deformity of the spine.

According to the above inclusion and exclusion criteria, 8 patients with thoracolumbar spinal TB who received postural drainage with intervertebral space as the treatment center in our department from June 2012 to August 2019 were included in this study, including 5 males and 3 females. Approved by the 960th hospital of PLA, this study involved accessing data for research purposes on November 2, 2022. Each author certifies that all investigations were conducted in accordance with ethical principles. The participant involved in the study gave their informed consent and signed an informed consent form. The average age was (42.14 ± 17.54) years old (20–68 years old). Eight cases were all newly diagnosed patients, and the course of the disease was 1 to 24 months, with an average of 7.75 ± 10.19 months; 8 cases had obvious lower back pain, lower limb pain, weakness, numbness, 5 cases with urinary and bowel dysfunction and other neurological symptoms; 1 case was combined with diabetes mellitus. Imaging examination revealed: 2 cases of thoracolumbar and 6 cases of lumbar spine; 6 cases of single-segment involvement and 2 cases of double-segment involvement; a total of 4 thoracic vertebrae, 12 lumbar vertebrae and 2 sacral vertebrae. The general information of the patients is shown in Table 1.

**Table 1:** Materials.

No.	Gender	Age	Temperature	Blood glucose level	Course of disease (month)	Affected segment	Location of abscesses outside the space				
							Anterior vertebra	Paravertebral	Intraspinal	Posterior lamina	Psoas major
1	M	21	37.5	3.97	1	T11-L1	**+**	**+**	**−**	**−**	**+**
2	F	36	36.9	6.01	1	L4-L5	**+**	**+**	**−**	**−**	**+**
3	M	53	36.5	10.86	1	L2-L3	**+**	**+**	**−**	**−**	**+**
4	M	20	38.3	4.68	6	T11-L1	**+**	**+**	**−**	**−**	**+**
5	F	47	37.7	5.45	1	L2-L3	**+**	**+**	**−**	**+**	**−**
6	M	30	37.4	4.51	4	L5-S1	**+**	**+**	**+**	**−**	**+**
7	M	68	36.3	4.63	24	L5-S1	**+**	**+**	**−**	**+**	**+**
8	F	50	36.2	4.59	24	L3-L4	**+**	**+**	**+**	**−**	**+**

### 2.2. Treatment

The admission chemotherapy regimen was standardized antituberculosis treatment according to the principles of early, regular, appropriate, full course, and combined medication. The patients received isoniazid (300 mg/d), rifampicin (600 mg/d), and pyrazinamide (750 mg/d), ethambutol (750 mg/d) quadruple (HRZE) chemotherapy regimen, take it on an empty stomach every morning, and regularly review blood routine, erythrocyte sedimentation rate (ESR), and C-reactive protein, and pay attention to the clinical efficacy of antituberculosis drugs. According to the results of X-ray, computerized tomography (CT), and magnetic resonance imaging, a postural drainage scheme with the intervertebral space as the treatment center was designed. Under local anesthesia, the transforaminal endoscope is used to assist percutaneous puncture and catheterization to reach the center of the infection and destruction of the intervertebral space, and the fluid can be immediately flushed to ensure smooth postural drainage. For patients with difficult volume, the step of directly extracting pus by percutaneous puncture can be added in time for treatment. Regularly review blood routine, ESR, CRP, and drainage fluid to check acid-fast staining and bacterial culture. When ESR and CRP drop to less than half of the peak value within a week, and there is no obvious drainage fluid for 3 consecutive days under the condition of changing body position, the drainage tube can be removed. During the treatment period, the patients were instructed to maintain a high-protein, high-vitamin, and high-calorie diet.

### 2.3. Efficacy evaluation

During the treatment period, patients were required to undergo regular outpatient review at 1, 3, 6, 12, and 18 months, and then every 6 months. The clinical efficacy of antituberculosis treatment was evaluated according to ESR and CRP; the effect of postural drainage was evaluated according to imaging examination; the pain degree of the patients was evaluated by the pain Visual Analog Scale (VAS) score; the neurological function American Spinal Cord Injury Association classification and Oswestry functional index were used to evaluate the neurological recovery after treatment.

## 3. Result

**Figure 1.**
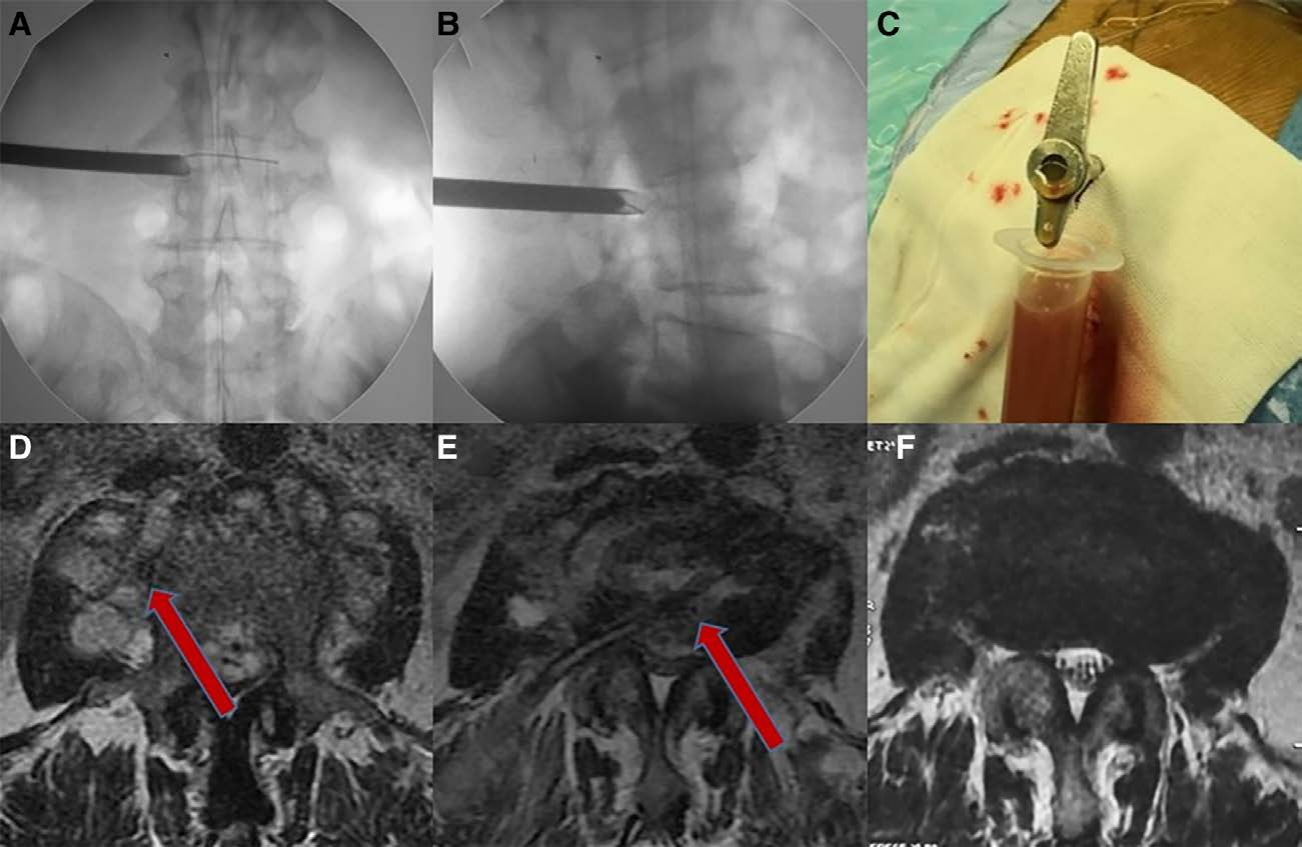
(A) Foraminal endoscopy-assisted lesion removal and cannulation. (B) The spontaneous outflow of tuberculous pus during the operation indicates that the pressure of the abscess in the intervertebral space is too high. (C) The L5/S1 space and the right psoas major muscle abscess formed. The arrows indicate that the psoas abscess comes from the perfusion dissemination of the intervertebral space. (D) MRI during postural drainage treatment, the drainage tube indicated by the arrow is in the intervertebral space and fixed in place. (E) Eighteen months after the operation, the MRI shows that the lesion is still, the abscess disappeared. MRI = magnetic resonance imaging.

**Figure 2.**
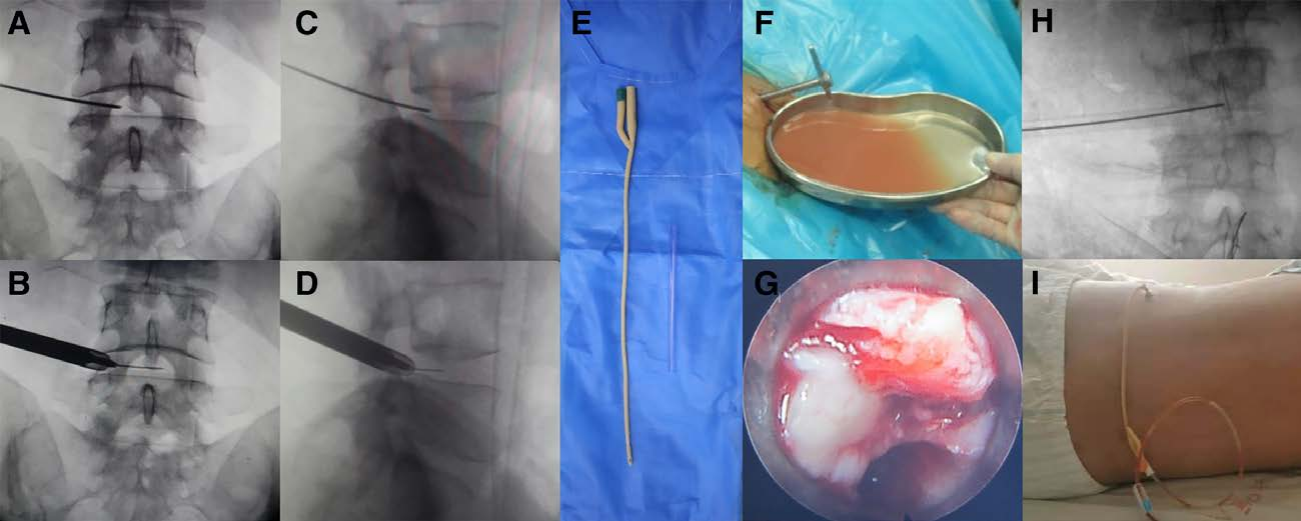
(A–D) The foraminal microscope has reached the infection space and the lesions are cleared. (F and G) A large number of tuberculosis abscesses are seen under the microscope, the pressure in the infection space is too high, and part of the pus flows out of the channel. (E) The common urinary catheter was transformed into a postural drainage tube for use. (H) The postural drainage tube (indicated by the arrow) was sent to the infection space with the help of a C-arm and a transforaminal mirror positioning guide wire. (I) The external photo after the catheter placement.

The operation time was 35 to 65 minutes, and the average operation time was 44.38 ± 10.50 minutes. The blood loss was 5 to 10 mL, and the average blood loss was 6.88 ± 2.59 mL. The follow-up period ranged from 36 to 122 months, with an average of 78.63 ± 34.45 months, and the standard antituberculosis drug treatment was carried out for 18 months. The average catheter drainage time was 13.25 ± 4.95 days, and the average total drainage volume was 281.25 ± 167.69 mL. Compared with the time of admission, after 7 days of postural drainage, the VAS score and ESR of the patients were significantly decreased, and the CRP was significantly increased, and there was a statistically significant difference between before and after (*P* < .05). At the last follow-up, the VAS score of the patient was 1.4 ± 0.52, the Oswestry functional index was 6 ± 4, the ESR was 8.63 ± 2.07 mm/h, and the CRP was 5.74 ± 2.94 mg/L, which were significantly lower than those at the time of admission. Statistical difference (*P* < .05). The above related results are shown in Table 2. At the last follow-up, all patients had no symptoms of TB recurrence such as low back pain, and imaging examinations showed that spinal TB was cured. A typical case is shown in Figure 1, and a schematic flow of catheter placement is shown in Figure 2.

**Table 2:** Evaluation of treatment effect of patients.

No.	Follow-up time (month)	Before drainage						7 days after drainage			Last follow-up				First diversion flow (mL)	Total diversion flow (mL)	Drainage time (d)
		VAS score	CRP	ESR	ASIA	Oswestry functional index	VAS score	CRP	ESR	VAS score	CRP	ESR	ASIA	Oswestry functional index			
1	122	8	42.6	48	E	56	3	104.34	17	1	7.12	12	E	10	50	685	20
2	118	8	16.9	55	E	54	4	66.17	24	1	4.49	7	E	4	65	205	7
3	114	7	46.1	18	E	53	5	91	20	2	11.1	11	E	13	55	170	13
4	91	8	44.9	43	D	34	2	104.29	20	1	2.97	7	E	5	80	290	14
5	61	8	12.6	84	E	78	3	73	13	1	8.31	9	E	4	60	190	16
6	49	8	52.7	30	D	66	5	102.14	11	2	4.78	9	E	3	50	260	19
7	38	8	4.56	28	E	46	4	85.9	15	1	2.17	6	E	1	75	240	9
8	36	8	8.46	39	E	61	6	63	7	2	5	8	E	8	110	210	8
Average value	78.63	7.88	28.6	43.13		56	4	86.23	15.88	1.38	5.74	8.63		6	68.13	281.25	13.25

ASIA = American Spinal Cord Injury Association, CRP = C-reactive protein, ESR = erythrocyte sedimentation rate, VAS = Visual Analogue Scale.

## 4. Discussion

The abscess drainage methods of spinal TB have been reported in many literatures (Table 3), mainly CT and percutaneous endoscopy-assisted abscess drainage and lavage and local chemotherapy. At present, a number of studies have reported that minimally invasive or endoscopic techniques such as CT-guided puncture and drainage have been used in the treatment of spinal TB and achieved certain clinical curative effects. Researchers including Zhang Zehua,^[15]^ Li Tianqing,^[16]^ Zhang Shengxun,^[17]^ Hou Xiaohua,^[18]^ and Tinnakorn Pluemvitayaporn^[19]^ reported that the use of CT-guided puncture and catheterization to drain psoas abscesses and local chemotherapy can alleviate the progression of spinal TB and can effectively cure lesions. Yin Xinhua^[20]^ and others reported that only 2 cases of recurrence were treated in 27 children with spinal TB by CT-guided puncture and catheter drainage and local irrigation. Yang Huadong et al^[21]^ reported that 31 cases of thoracolumbar TB with intraspinal abscess were treated with minimally invasive small incision interlaminar decompression combined with local chemotherapy and achieved satisfactory results. Zhang Zhifa^[22]^ and others retrospectively analyzed 106 patients with spinal TB who were treated with CT-guided catheter drainage and percutaneous infusion chemotherapy, and also proved that minimally invasive surgery is effective in the treatment of spinal TB. In our group of cases, the operation time, blood loss, and drainage time are better than those reported in the literature, and 8 patients have no long-term complications such as recurrence.

**Table 3:** Review of relevant literature.

Reference	Number of cases	Average age (years)	Drainage mode	Operation time (minutes)	Blood loss (mL)	Drainage time (days)	Follow-up time (month)
Zehua Zhang^[15]^	48	29.44 ± 12.40	CT-guided percutaneous catheter drainage	53.65 ± 18.00	15.44 ± 13.72	60 ± 121.90	35.88 ± 13.74
Xinhua Yin^[16]^	27	4.55 ± 2.02	CT-guided modified percutaneous catheter drainage	4.55 ± 2.02	Not mentioned	24.89 ± 3.59	31.00 ± 13.94
Shengxun Zhang^[17]^	11	43.91 ± 4.97	CT-guided continuous catheterization drainage	100.80 ± 10.2	618.36 ± 183.37	12.55 ± 1.19	Not mentioned
Huadong Yang^[18]^	31	53.3	Minimally invasive spinal decompression with local chemotherapy	80	90	Not mentioned	37
Tianqing Li^[19]^	45	36.45	CT-guided minimally invasive catheter drainage	Not mentioned	Not mentioned	120	60
Tinnakorn Pluemvitayaporn^[20]^	29	44.03 ± 11.02	CT-guided percutaneous catheter drainage	Not mentioned	Not mentioned	17	36
Zhifa Zhang^[21]^	106	Not mentioned	CT-guided percutaneous catheter drainage and percutaneous catheter infusion chemotherapy	Not mentioned	Not mentioned	Not mentioned	86.52 ± 37.8
Xiaohua Hou^[22]^	27	56 ± 14	CT-guided percutaneous focal catheter infusion	Not mentioned	Not mentioned	29 ± 11	19 ± 4
Our’s	8	42.14 ± 17.54	Intervertebral space centered postural drainage	44.38 ± 10.50	6.88 ± 2.59	13.25 ± 4.95	78.63 ± 34.45

Some literature data on the treatment of spinal tuberculosis with catheter drainage technology were reported, including drainage mode, operation time, blood loss, drainage time and follow-up time, in the past 10 years.

It has been reported that the drainage core of irrigation and drainage is the paraspinal, psoas major or iliopsoas muscle with large abscess,^[19]^ which is prone to obstruction of the drainage tube, and has no effect on the “bacteria nest”-inadequate drainage of intervertebral space lesions. Ineffective, it is more difficult for spinal abscesses, which increases the drainage time and treatment time, and is also the source of recurrence^[22]^; *M tuberculosis* uses the intervertebral space as a nest to form a cold abscess, which breaks through the natural space around and spreads to the surrounding area. Adjacent vertebral body, psoas muscle, spinal canal, paravertebral, etc. The spread of the abscess proves that the pressure in the abscess cavity is too high, and it is our theoretical basis to reduce the local and peripheral pressure by draining the indwelling tube. Drainage centered on the intervertebral space does not require re-drainage of the psoas abscess, and the intervertebral space lesions and the psoas major lesions are interconnected.^[23]^ Under the intervention of postural drainage, the abscess cavity in the intervertebral space was relatively low pressure, and the pus in the rest of the body returned to the “bacterial nest.” Therefore, we believe that the focus of lesion removal and drainage is the intervertebral space, and the intervertebral space forms a cavity after local destruction, which reduces the risk of tube blockage and enables sustainable drainage. Our group of patients had been drained for a total of 13.25 ± 4.95 days, and no blockage occurred until the time of extubation. We use foraminal debridement + drainage to treat primary lesions, which can effectively curb bacterial reproduction. For some patients with larger abscesses, this method can also be adopted: the primary lesions of the intervertebral space are treated by continuous drainage, and the pus in the adjacent compartment is treated by reflux, puncture, and natural absorption.

Patients in this study all have one thing in common, the basic physique is good, and can walk independently, with no vertebral instability, deformity, mild neurological symptoms. Therefore, we adopt the minimally invasive method of local anesthesia, which not only has little trauma, quick recovery, and little impact on the body’s immunity; but also because the patient has strong mobility, he can easily change the position, such as: head high, feet high, side lying, autonomous increase abdominal pressure, ground activities, and other methods to effectively enhance the drainage effect. In addition, *M tuberculosis* often destroys the anterior and middle column structures such as the vertebral body and intervertebral space, while the posterior column is relatively intact, which is also the basis for the formation of scoliosis and kyphosis.^[6]^ For patients with intervertebral space destruction, early intervention can be performed to prevent the destruction and aggravation of spinal deformity. Our point of view is to focus on the intervertebral space as the center. Foraminal debridement and drainage under early local anesthesia can control the disease, reduce the damage to normal tissues, and prevent spinal deformities.

All 8 cases of spinal TB had obvious local pain, destruction of intervertebral space and formation of cold abscess. We used transforaminal endoscope to place a postural drainage tube into the infection space eroded and destroyed by TB, and performed adequate postural drainage until the standard of withdrawal was reached. From the statistical results, it can be seen that after 7 days of drainage, the patient’s VAS score and ESR were significantly reduced, which indicates that rapid drainage to clear TB cold abscesses and reduce bacterial load is an effective way to relieve symptoms of TB poisoning. The data of CRP in Tianhou showed that CRP was significantly increased, indicating that after puncture and drainage of TB cold abscess, the symptoms of TB poisoning were rapidly relieved, and the body’s own immune system was fully activated, which promoted the ability to clear *M tuberculosis*. As of the last follow-up, the patient’s VAS score, Oswestry functional index, ESR, and CRP were all within the normal range.

## 5. Conclusion

To sum up, we believe that the center and focus of spinal TB treatment is to clear and adequately drain the intervertebral space, which can be treated with postural drainage of the lesions with the intervertebral space as the treatment center. All 8 patients in this group obtained good prognosis, but the number of cases in this study is small, and the detailed advantages and disadvantages of this treatment method need to be confirmed by further long-term observation and research with large samples and multi-center in the later stage.

## Author contributions

**Conceptualization:** Zhengqi Chang.

**Data curation:** Hao Xing, Jianhua Li.

**Funding acquisition:** Zhengqi Chang.

**Investigation:** Hao Xing, Jianhua Li.

**Writing – original draft:** Hao Xing.

**Writing – review & editing:** Zhengqi Chang.
